# IGFBP1^hi^WNT3A^lo^ Subtype in Esophageal Cancer Predicts Response and Prolonged Survival with PD-(L)1 Inhibitor

**DOI:** 10.3390/biology11111575

**Published:** 2022-10-27

**Authors:** Meichen Liu, Wanpu Yan, Dongbo Chen, Jiancheng Luo, Liang Dai, Hongsong Chen, Ke-Neng Chen

**Affiliations:** 1 Department of Thoracic Surgery I, Key Laboratory of Carcinogenesis and Translational Research (Ministry of Education), Peking University Cancer Hospital & Institute, No. 52, Fucheng Road, Haidian District, Beijing 100142, China; 2 Peking University People’s Hospital, Peking University Hepatology Institute, Beijing Key Laboratory of Hepatitis C and Immunotherapy for Liver Disease, No.11 Xizhimen South Street, Xicheng District, Beijing 100044, China; 3 Aiyi Technology Co., Ltd., Room 1004, Building 3, Greenland Qihang, Biomedical Industry Base, Daxing District, Beijing 102629, China

**Keywords:** esophageal cancer, immunotherapy, WNT signaling, IGFBP1, WNT3A

## Abstract

**Simple Summary:**

The lack of precision biomarkers hinders the development of individualized immunotherapy for esophageal cancer (ESCA) patients. The activation of WNT signaling has proved to be associated with the primary resistance to immunotherapy. Therefore, our study aims to develop efficient biomarkers based on WNT signaling to guide ESCA immunotherapy. In the TCGA cohort with 196 cases and our BJCH cohort with 95 cases, we successfully constructed an IGFBP1^hi^WNT3A^lo^ signature and validated its correlation with the poor prognosis of ESCA patients. Moreover, in 2 GEO cohorts with a total of 68 cases and our BJIM cohort with 21 cases, we verified that IGFBP1^hi^WNT3A^lo^ ESCA patients had a good response and prognosis with immunotherapy. Our findings indicate that the IGFBP1^hi^WNT3A^lo^ signature was a potential immunotherapeutic biomarker for ESCA.

**Abstract:**

PD-(L)1 inhibitor could improve the survival of locally advanced esophageal cancer (ESCA) patients, but we cannot tailor the treatment to common biomarkers. WNT signaling activation was associated with primary resistance to immunotherapy. In this study, we used our two clinical cohorts (BJCH *n* = 95, BJIM *n* = 21) and three public cohorts to evaluate and verify a new immunotherapeutic biomarker based on WNT signaling in ESCA patients. Our findings showed that WNT signaling-related genes stratified TCGA patients into Cluster 1, 2, and 3, among which, Cluster 3 had the worst prognosis. The most up- and down-regulated genes in Cluster 3 were IGFBP1 and WNT3A. Further analysis validated that IGFBP1^hi^WNT3A^lo^ ESCA patients had significantly poor RFS and OS in the TCGA and BJCH cohorts. Interestingly, IGFBP1^hi^WNT3A^lo^ patients had a good response and prognosis with immunotherapy in three independent cohorts, exhibiting better predictive value than PD-L1 expression (signature AUC = 0.750; PD-L1 AUC = 0.571). Moreover, IGFBP1^hi^WNT3A^lo^ patients may benefit more from immunotherapy than standard treatment (*p* = 0.026). Immune cell infiltration analysis revealed a significant increase in DC infiltration in IGFBP1^hi^WNT3A^lo^ patients post-immunotherapy (*p* = 0.022), which may enhance immune response. The IGFBP1^hi^WNT3A^lo^ signature could predict patients who benefited from PD-(L)1 inhibitor treatment and may serve as a biomarker in ESCA.

## 1. Introduction

Esophageal cancer (ESCA) is ranked eighth for cancer incidence and sixth for cancer mortality in the world [1], whereas China has a higher incidence and mortality rate of ESCA [2]. Neoadjuvant therapy followed by surgery is the standard treatment for locally advanced esophageal cancer, but the 5-year overall survival rate is still unsatisfactory [3].

Immunotherapy, represented by PD-1 or PD-L1 inhibitor, offers new hope for advanced esophageal cancer. In the randomized, advanced-stage, second-line phase III trial (Keynote-181) [4], overall survival (OS) was prolonged in esophageal cancer patients treated with pembrolizumab compared with those treated with platinum-based chemotherapy (median 9.3 vs. 6.7 months; *p* = 0.0074). The subsequently published global, randomized, placebo-controlled phase III trial (CheckMate-577) found nivolumab significantly prolonged median disease-free survival (DFS) in patients with locally advanced esophageal or gastresophageal junction cancer treated with pre-operative chemoradiation (median nivolumab vs. placebo: 22.4 vs. 11.0; *p* < 0.001) [5]. In addition, several clinical trials aiming to evaluate the efficacy of neoadjuvant immunotherapy for esophageal cancer are underway, including NICE, Keystone-001, Keystone-002, FRONTiER, and HCHTOG1909 [6,7,8].

Although immunotherapy improves the survival of esophageal cancer, there are still some patients who may not benefit from immunotherapy [9]; therefore, biomarkers that can predict the efficacy of immunotherapy to guide clinical practice are urgently needed. The most widely used biomarker is PD-L1 expression, but it could not accurately predict the outcome of patients who underwent the immunotherapy; for example, in the Keynote-062 trial, it was found that PD-L1 expression was not associated with OS or progression-free survival (PFS) and that some patients with positive PD-L1 expression may not benefit from immunotherapy [10]. Tumor mutation burden (TMB) is another biomarker used in pan-cancer. The multicohort, open-label, phase II KEYNOTE-158 trial verified that patients with high TMB had better outcome after accepting immunotherapy [11]. In addition, commonly used biomarkers have tumor-infiltrating lymphocyte (TIL), microsatellite instability/mismatch repair gene defects (MSI/dMMR) and T-cell receptor clonality [12,13]. However, as with PD-L1 expression, some positive patients may not benefit. Therefore, the development of new biomarkers is important to promote individualized treatment for patients with esophageal cancer.

WNT signaling is oncogenic signaling which is thought to be strongly associated with immunotherapy efficacy [14]. Generally, WNT signaling is divided into canonical (β-catenin-dependent) or non-canonical (β-catenin-independent) signaling. The correlation between WNT signaling activation and resistance to immune checkpoint blockade, such as CTLA-4 and PD(L)-1, was identified in melanoma [15,16] and hepatocellular carcinoma (HCC) [17]. Meanwhile, canonical WNT signaling activation is negatively correlated with T-cell infiltration in the pan-cancerous tumor immune microenvironment [18]. Patients with CTNNB1 (coding β-catenin) mutation may have lower PFS when they have received immunotherapy (*p* < 0.001) [19]. These results suggest that the correlations between WNT signaling and immunotherapy effects are worth evaluating. Nevertheless, there are few studies on esophageal cancer. The WNT signaling contains numerous components and is subject to many crosstalk mechanisms. Among them, WNT3A, a member of the WNT ligand family, is a signaling protein to activate the canonical WNT signaling [20]. WNT3A has been implicated in oncogenesis processes, including regulation of cancer cell differentiation and proliferation [21]. IGFBP1 is the first member of the IGFBP family, which circulates in the plasma and binds both IGF I and II, prolonging their half-life and regulating cell migration and metabolism [22,23]. However, there is no research on the relationship between IGFBP1 and WNT signaling. The IGFBP family protein IGFBP2 has been shown to upregulate the β-catenin expression [24].

In this study, we explore the relationship between WNT signaling and the efficacy of immunotherapy in esophageal cancer, aiming to find new biomarkers that would guide esophageal cancer immunotherapy.

## 2. Materials and Methods

### 2.1. Analysis of 3 Public Esophageal Cancer Cohorts from TCGA and GEO

The WNT signaling gene set was obtained from the Gene Set Enrichment Analysis (GSEA) (https://www.gsea-msigdb.org/, accessed on 15 September 2022). Gene expression and mutation data and clinical information for an esophageal cancer cohort (*n* = 196) were obtained from The Cancer Genome Atlas (TCGA) (https://portal.gdc.cancer.gov/, accessed on 15 September 2022). The esophageal cancer immunotherapy cohorts were collated from Gene Expression Omnibus (GEO) (https://www.ncbi.nlm.nih.gov/gds, accessed on 15 September 2022). Samples from the GSE183924 cohort (*n* = 37) were from a phase II clinical trial [25]. This trial enrolled US patients with locally advanced esophageal adenocarcinoma treated with neoadjuvant radiotherapy combined with surgery followed by sequential 1-year treatment with PD-L1 therapy (durvalumab). Samples from the GSE165252 cohort (*n* = 37) were from the phase II PERFECT trial [26]. This trial enrolled US patients with resectable esophageal adenocarcinoma treated with neoadjuvant PD-L1 therapy (atezolizumab) comminated with radiotherapy followed by radical resection. Patient response to treatment was assessed according to the Response Evaluation Criteria in Solid Tumors (RECIST) 1.1 guidelines.

### 2.2. Analysis of 2 Clinical Esophageal Cancer Cohorts from BJCH and BJIM

Both the Peking University Cancer Hospital (BJCH) and Peking University Cancer Hospital Immunotherapy (BJIM) cohorts were obtained from the prospective maintained clinical database of the first Department of Thoracic Surgery, Peking University Cancer Hospital and Institute. The BJCH cohort included 95 consecutive patients with esophageal cancer who underwent radical resection from November 2018 to July 2020. Tumor and adjacent normal mucosa tissue samples were collected for paraffin embedding. The BJIM cohort included 21 consecutive patients with esophageal squamous cancer who received neoadjuvant PD-1 (Toripalimab) or PD-L1 (ZKAB001, an anti-PD-L1 antibody) combination chemotherapy and radical resection from January 2021 to August 2021. Fresh tumor and adjacent normal mucosa tissue samples were collected and stored at −80 °C. Immunotherapy efficacy was assessed according to iRECIST 1.1 guidelines [27]. All patients were divided into two groups: responder, including complete response (CR)/partial response (PR), and non-responder, including stable disease (SD)/progressive disease (PD). Clinical staging was performed according to Union for International Cancer Control (UICC)/American Joint Committee on Cancer (AJCC) 8th edition TNM staging guidelines for esophageal cancer. All patients were diagnosed as having esophageal cancer by 2 experienced pathologists, and PD-L1 expression (22C3) was assessed in the BJIM cohort.

### 2.3. Immunohistochemistry Analysis of the BJCH Cohort

After deparaffinization, rehydration, blocking of endogenous peroxidases, antigen retrieval and blocking, tissue sections were incubated with primary antibodies at 4 °C overnight. After brief washes in PBS, corresponding secondary antibodies were added and incubated at 37 °C for 20 min. The samples were visualized after DAB staining. The primary antibodies and dilutions used were as follows: anti-IGFBP1 (Santa Cruz, sc-55474, 1:100) and -WNT3A (Abcam, ab219412, 1:500). The staining intensity was scored on a scale of 0–3, with 0, no staining; 1, weak staining; 2, moderate staining; and 3, strong staining. The staining extensity was defined as follows: 0, no positive cell; 1, 1% to 25%; 2, 26% to 50%; and 3, 50% to 100%. The final composite score was the sum of the intensity and extensity scores. The composite scores of 0–2 were defined as low expression, and scores of 3–6 were defined as high expression.

### 2.4. Next-Generation Sequencing and Analysis of the BJIM Cohort

General transcriptome sequencing was performed by Tianjin Novogene Biotechnology Co. Total RNA was extracted from tumor and paired samples using TRIzol reagent, and an Agilent 5400 was used to detect the concentration, total amount, and integrity of the RNA. Based on quality control results, the total amount of RNA in all samples was ≥10 μg, and the integrity was ≥7.0, meeting the requirements for sequencing. The Nova-PE150 platform was used for sequencing. RNA-seq FASTQ files were used to quantify gene expression with Kallisto [28] as gene-specific counts, transcripts per million (TPM), and normalized log2(TPM+1). We used TPM for the ensuing analysis.

### 2.5. Grouping and Marker Gene Mining Based on WNT Signaling

Unsupervised hierarchical clustering was used to stratify the cohort from TCGA (*n* = 196) to obtain 55 WNT signaling-related genes and compare expression differences among subtypes. The median was taken as the cut-off value, and two pairs of significantly upregulated and significantly downregulated genes were used as the basis for typing. The combination with the highest similarity to the hierarchical clustering typing results was selected to construct the signature. The predictive value of the signature for prognosis and immunotherapy efficacy was assessed using survival analysis and iRECIST 1.1 guidelines. The predictive effect was compared with that of PD-L1 expression using receiver operating characteristic (ROC) curve. PD-L1 expression in samples was described using the combined positive score (CPS), which refers to the number of PD-L1-stained cells/total tumor cells × 100. PD-L1 (CPS) ≥10 was used as the cut-off value for typing [10] and was defined as positive expression. Multifactorial Cox regression analysis incorporated age, gender, smoking history, drinking history, TNM stage, PD-L1 expression, IGFBP1 expression, WNT3A expression and the IGFBP1^hi^WNT3A^lo^ signature.

### 2.6. Gene Set Enrichment Analysis

Changes in WNT signaling expression between esophageal cancer subtypes were compared using single-sample gene set enrichment analysis (ssGSEA), and the normalized enrichment score (NES) was calculated. Gene Ontology (GO) and Kyoto Encyclopedia of Genes and Genomes (KEGG) were obtained from Molecular Signatures Database (MSigDB). *p* < 0.05, false discovery rate (FDR) < 0.25, and |NES| > 1 were selected as the criteria for significant enrichment in GO and KEGG analyses. A heatmap was used to demonstrate enrichment.

### 2.7. Immune Cell Infiltration and Molecular Characterization

The tumor mutational burden (TMB) refers to the total number of somatic mutations per million bases, including base substitutions and insertions/deletions. Intratumor heterogeneity (ITH) was quantified using the MATH method, and mutational features were annotated using COSMIC v2. MCP-counter assesses immune cell infiltration within tumors, and MCP-counter [29] could quantify the absolute abundance of eight immune cell types and two stromal cell types in tissues from gene expression data. B cell receptor (BCR) and T cell receptor (TCR) richness and diversity data were obtained from the Supplemental Material of the article by Thorsson et al. [30] Differences in the expression of immunoregulatory genes, such as PD-L1, PD-L2, and TGF-β1were assessed using the cohort from TCGA.

### 2.8. Statistical Analysis

Measures are statistically described using the mean ± standard deviation. The student’s t-test or the nonparametric rank-sum test was used for statistical comparisons. Count data were statistically compared using the X^2^ test or Fisher’s exact test. The Kaplan–Meier method was employed for survival analysis and the log-rank test to determine significant differences in survival. Cox regression analysis was applied for independent predictor tests. OS was defined as the time from the date of surgery to death from any cause. RFS was defined as the time from the date of surgery to disease relapse or death from any cause. ROC curves were used to calculate the area under the curve (AUC) for comparisons of predictive efficiency. Sample sizes were calculated using the Proc Power procedure in SAS software (version 9.4). The sample sizes of cohorts were suitable for analysis. All statistics were performed using a two-sided test, with *p* < 0.05 as the criterion for a significant difference.

## 3. Results

### 3.1. Patients with Esophageal Cancer Could Be Stratified into 3 Clusters Based on WNT Signaling

**Figure 1:**
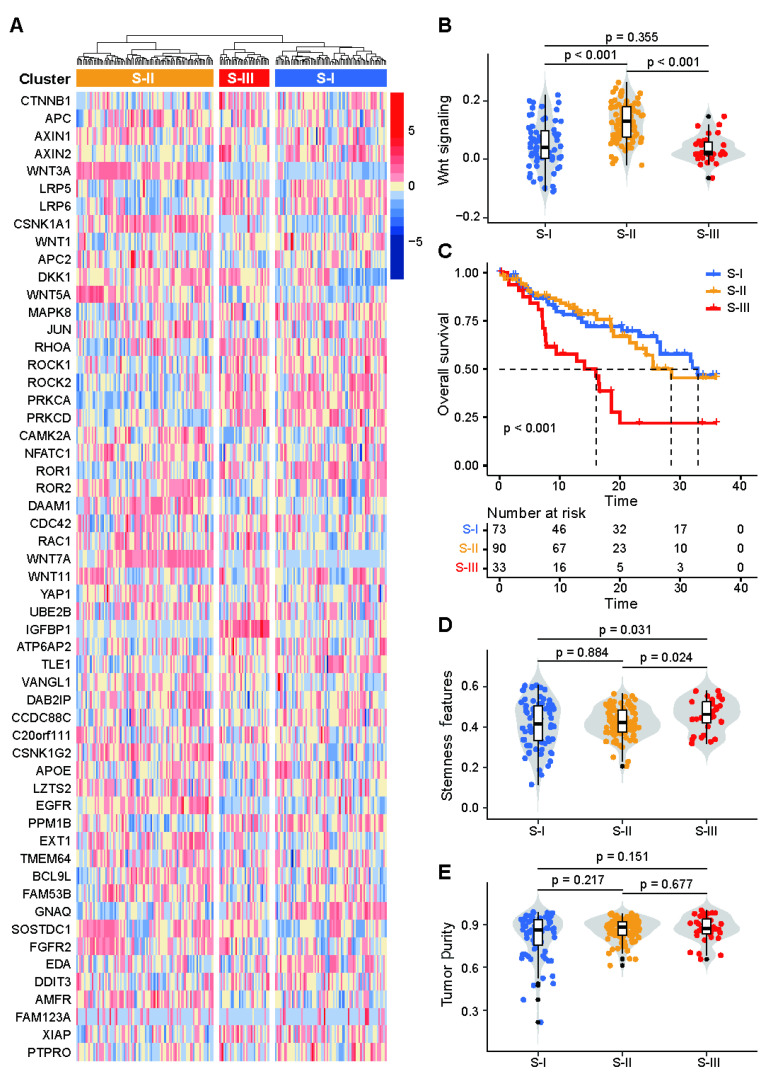
Tumor clustering based on WNT signaling. (**A**) WNT signaling-related genes were used for unsupervised hierarchical clustering to classify the 196 patients in the cohort from TCGA into 3 clusters: Clusters 1, 2 and 3; (**B**) ssGSEA revealed significant activation of WNT signaling in Cluster 2, significant inhibition in Cluster 3, and no significant change in Cluster 1; (**C**) Kaplan–Meier survival analysis suggested that Cluster 3 had the worst prognosis and Clusters 1 and 2 had a similar prognosis; (**D**) Stemness feature analysis showed that Cluster 3 had the highest stemness features and Clusters 1 and 2 had similar stemness features; (**E**) No difference in tumor purity between clusters was found.

Unsupervised hierarchical clustering analysis revealed that 55 WNT signaling-related genes could classify the patients in the cohort from TCGA (*n* = 196) into 3 clusters (Figure 1A). ssGSEA results showed significant activation of WNT signaling in Cluster 2, significant inhibition of WNT signaling in Cluster 3, but no significant changes in WNT signaling in Cluster 1 (Figure 1B).

The prognosis of patients within Clusters 1 and 2 was similar (median 13.6 vs. 13.0 months, *p* > 0.05), and there was no difference in stemness features between these two clusters (*p* = 0.884) (Figure 1C,D). Compared with Cluster 1 and 2, we found that patients within Cluster 3 had the worst prognosis (median 9.4 months, *p* < 0.001) and the highest number of stemness features (*p* < 0.05). Tumor purity is the systematic error that probably has the greatest impact on results, and there was no difference in tumor purity among Clusters 1, 2 and 3 after testing (*p* > 0.05) (Figure 1E). In our ensuing analysis, we focused on the Cluster 3.

Furthermore, to better explore the genomic characteristics of patients within Cluster 3, we combined Clusters 1 and 2 for subsequent analysis.

### 3.2. The Cluster 3 of Marker Genes Are IGFBP1^hi^ and WNT3A^lo^

**Figure 2:**
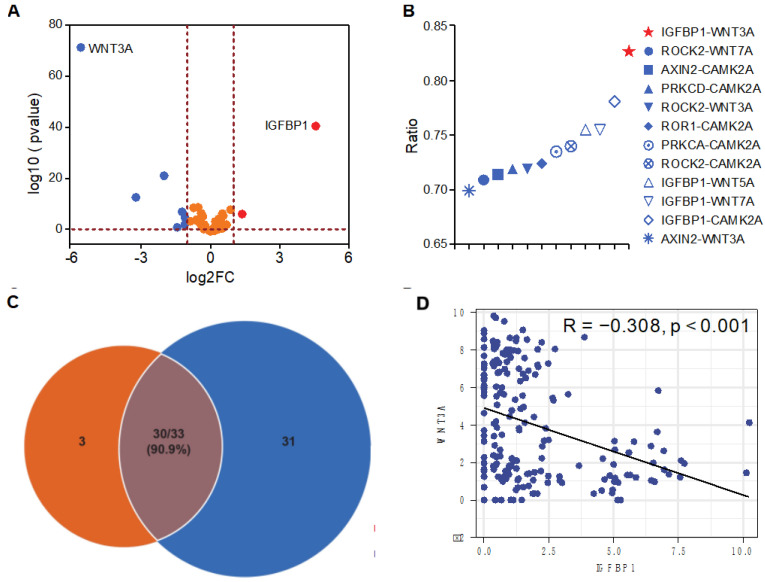
Potential predictors of immunotherapy efficacy. (**A**) Volcano plot showing that IGFBP1 and WNT3A were most significantly differentially expressed; (**B**,**C**) the consistency of the upregulated and downregulated combination subtype was compared with Clusters 1, 2 and 3; (**D**) correlation analysis of IGFBP1 and WNT3A expression.

First, we compared the differential expression of 55 WNT signaling-related genes between Cluster 3 and Cluster 1+2 (Tables S1 and S2) and found that genes such as IGFBP1, AXIN2, PRKCA, PRKCD, ROR1 and ROCK2 were significantly upregulated, but that WNT3A, WNT5A, WNT7A and CAMK2A were significantly downregulated in Cluster 3. Among the significant genes, the expressions of IGFBP1 and WNT3A were the most significantly different (Figure 2A). Then, an upregulated gene and a downregulated gene from 55 WNT signaling-related genes were combined to reclassify the TCGA cohort and further compared the consistency with the results of unsupervised hierarchical clustering. The overall consistency ranged from 69.9% to 82.7%, with the highest consistency of IGFBP1-WNT3A (82.7%), followed by IGFBP1-CAMK2A (78.1%) and IGFBP1-WNT7A (75.5%). Moreover, the IGFBP1^hi^WNT3A^lo^ subtype was consistent with the Cluster 3 up to 90.9% (Figure 2B,C). Further analysis revealed that IGFBP1 expression and WNT3A expression correlated significantly negatively (*p* < 0.001) (Figure 2D). Other results of correlation analysis between significant upregulated and downregulated genes were in Table S3. Therefore, two subtypes, IGFBP1^hi^WNT3A^lo^ and non IGFBP1^hi^WNT3A^lo^, were classified according to the expression of IGFBP1 and WNT3A. Patients in the non-IGFBP1^hi^WNT3A^lo^ subtype included patients with IGFBP1^hi^WNT3A^hi^, IGFBP1^lo^WNT3A^hi^ or IGFBP1^lo^WNT3A^lo^ status.

### 3.3. Molecular Characterization of the IGFBP1^hi^WNT3A^lo^ Subtype

**Figure 3:**
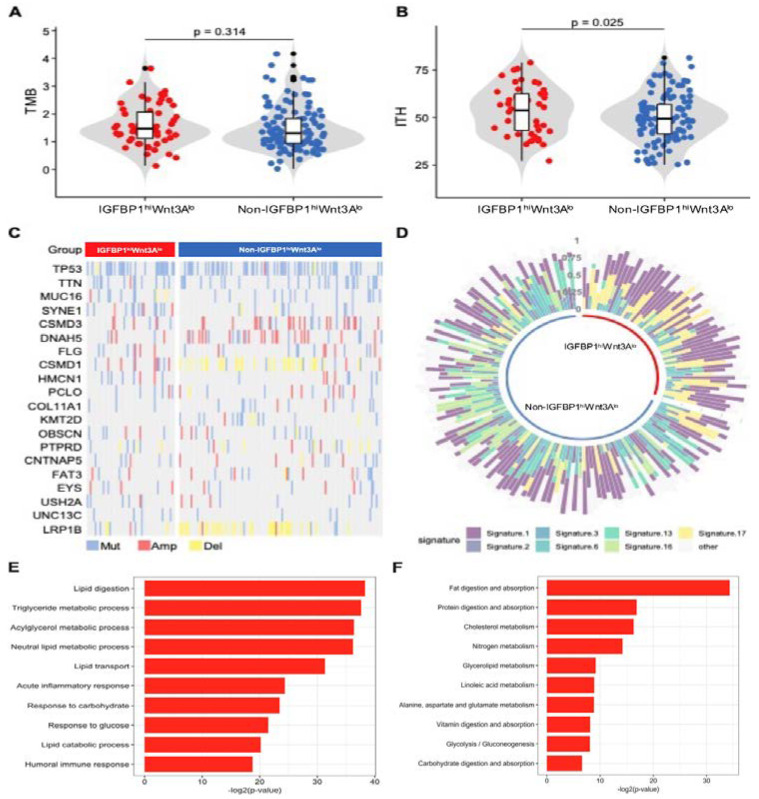
Molecular characterization of subtypes. (**A**,**B**) Violin plots illustrating TMB and ITH differences between subtypes in the cohort from TCGA; (**C**) comparison of mutated genes between subtypes sorted by mutation frequency; (**D**) mutation characteristics between subtypes in the cohort from TCGA; (**E**,**F**) GO and KEGG analyses used significantly upregulated differential expression genes. The results revealed activation of immune-related pathways and imbalance of energy metabolism in the IGFBP1^hi^WNT3A^lo^ subtype.

First, our calculations indicated no significant difference in TMB between the IGFBP1^hi^WNT3A^lo^ subtype and the non-IGFBP1^hi^WNT3A^lo^ subtype (*p* = 0.428). Further analysis showed a significant increase in ITH in the IGFBP1^hi^WNT3A^lo^ subtype (*p* = 0.025) (Figure 3A,B). We then ranked the genes by mutation frequency. TP53 mutation was the gene with the highest mutation frequency, followed by TTN and MUC16. Several gene mutations have now been shown to be associated with immunotherapy response [31]. In the IGFBP1^hi^WNT3A^lo^ subtype, the frequency of MUC16 mutation was higher (16.4% vs. 10.4%) (Figure 3C). Finally, using COSMIC v2 to annotate the mutation signature (Figure 3D), we found a significant increase in Signature 1 and Signature 17 in the IGFBP1^hi^WNT3A^lo^ subtype. Signature 1 is the result of an endogenous mutational process initiated by spontaneous deamination of 5-methylcytosine, and its appearance is thought to be age-related. Signature 17 is not annotated in COSMIC v2.

In addition, GO enrichment analysis (Figure 3E) demonstrated significant upregulation of immune-related pathways, such as the acute inflammatory response pathway (*p* = 4.57 × 10^−8^) and humoral immune response pathway (*p* = 2.31 × 10^−6^). GO and KEGG enrichment analyses also identified multiple metabolism-related pathways with upregulated expression, including lipid metabolism, glycolysis/glycogenesis, and alanine, aspartate, and glutamate metabolism (Figure 3E,F).

### 3.4. The IGFBP1^hi^WNT3A^lo^ Subtype Correlates with Poor Prognosis in TCGA Esophageal Cancer Patients

**Figure 4:**
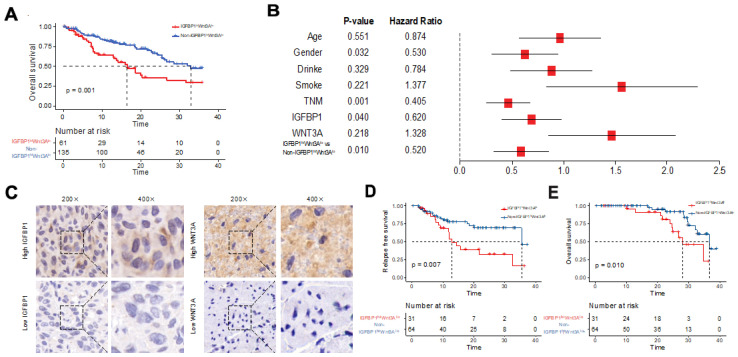
Relationship between the IGFBP1^hi^WNT3A^lo^ signature and prognosis of esophageal cancer. (**A**) Kaplan–Meier survival curve of OS in the cohort from TCGA; (**B**) forest plot showing the results of multifactorial Cox regression analysis the cohort from TCGA. The entry method was Forward:LR; (**C**) representative IHC for IGFBP1 and WNT3A; (**D**) Kaplan–Meier survival curve of RFS in the BJCH cohort; (**E**) Kaplan–Meier survival curve of OS in the BJCH cohort.

After reclassifying esophageal cancer patients in the TCGA cohort based on IGFBP1^hi^WNT3A^lo^, Kaplan–Meier survival analysis showed a poor prognosis for the IGFBP1^hi^WNT3A^lo^ subtype (median 9.1 months, *p* = 0.001) (Figure 4A). Multifactorial Cox regression analysis indicated that gender, TNM stage, IGFBP1 and the IGFBP1^hi^WNT3A^lo^ signature (*p* = 0.010; HR 0.520; 95% CI 0.380–0.803) was an independent predictor of prognosis in esophageal cancer (Figure 4B).

### 3.5. Validation That the IGFBP1^hi^WNT3A^lo^ Subtype Is Associated with Poor Prognosis in BJCH Esophageal Cancer Patients

We confirmed that the IGFBP1^hi^WNT3A^lo^ subtype was associated with poor prognosis in patients with esophageal cancer in the BJCH cohort (*n* = 95). Immunohistochemistry results (Figure 4C) showed 31 (32.6%) patients with the IGFBP1^hi^WNT3A^lo^ subtype and 64 (67.4%) patients with the non-IGFBP1^hi^WNT3A^lo^ subtype. In the comparison of clinical characteristics between two subtypes (Table S4), only family history was significantly different (*p* = 0.010), whereas there were no significant differences in sex, age, smoking history, drinking history, histological type, degree of differentiation, treatment methods or TNM stage (*p* > 0.05).

The results of survival analysis suggested poor OS (median 21.6 vs. 22.5 months, *p* = 0.010) and short RFS (median 9.4 vs. 13.1 months, *p* = 0.007) in IGFBP1^hi^WNT3A^lo^ patients with esophageal cancer (Figure 4D,E). Multifactorial Cox regression analysis (Table S5) confirmed that the IGFBP1^hi^WNT3A^lo^ signature was an independent predictor of prognosis (*p* = 0.046; HR 2.061; 95% CI 1.012–4.200).

### 3.6. The IGFBP1^hi^WNT3A^lo^ Subtype Is Associated with Good Immunotherapy Response and Prognosis in Esophageal Cancer

**Figure 5:**
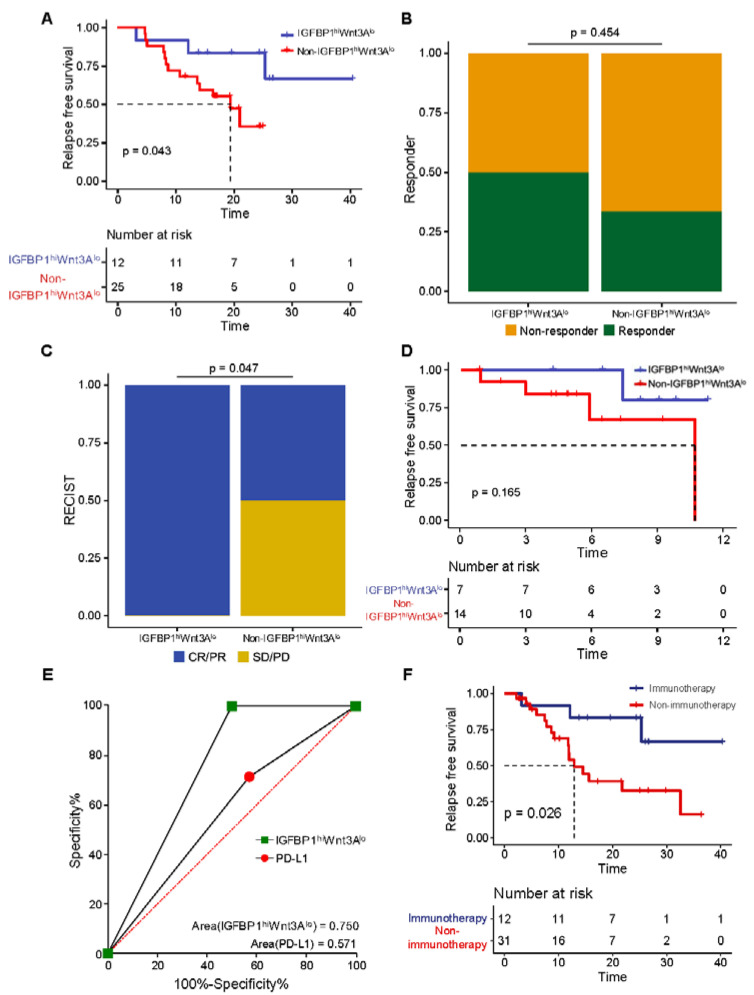
Relationship between the IGFBP1^hi^WNT3A^lo^ signature and immunotherapy of esophageal cancer. (**A**) Kaplan–Meier survival curve of RFS in the GSE183924 cohort; (**B**) bar chart showing the distribution of immunotherapy response among subtypes of the GSE165252 cohort; (**C**) bar chart showing the distribution of immunotherapy response among subtypes of the BJIM cohort; (**D**) Kaplan–Meier survival curve of RFS in the BJIM cohort; (**E**) ROC curve for assessing the predictive efficiency of the signature and PD-L1 expression; (**F**) comparison of RFS in IGFBP1^hi^WNT3A^lo^ subtype patients treated with or without immunotherapy.

We then tested the ability of the IGFBP1^hi^WNT3A^lo^ signature to predict immunotherapy response in patients with esophageal cancer in the GSE183924 cohort and the GSE165252 cohort. The GSE183924 cohort (*n* = 37) comprised 12 (32.4%) and 25 (67.6%) patients with the IGFBP1^hi^WNT3A^lo^ and non-IGFBP1^hi^ WNT3A^lo^ subtypes, respectively. After patients received immunotherapy, RFS was significantly prolonged in the IGFBP1^hi^WNT3A^lo^ subtype (median 25.2 vs. 16.6 months, *p* = 0.043) (Figure 5A). Meanwhile, this finding was validated in the GSE165252 cohort (Figure 5B), in which patients with the IGFBP1^hi^WNT3A^lo^ subtype had a greater response to immunotherapy than those with the non-IGFBP1^hi^WNT3A^lo^ subtype.

We further validated the above results in our BJIM cohort (*n* = 21) (Figure 5C). There were 7 (33.3%) patients with the IGFBP1^hi^WNT3A^lo^ subtype and 14 (66.7%) patients with the non-IGFBP1^hi^WNT3A^lo^ subtype. The comparison of clinical characteristics between the two subtypes revealed (Table 1) no significant differences in demographic and pathological characteristics, such as sex, age, smoking history, drinking history, family history, degree of differentiation, N stage and TNM stage (*p* > 0.05). Immunotherapy efficacy analysis showed that all (7/7) patients with the IGFBP1^hi^WNT3A^lo^ subtype responded to immunotherapy but that only 50% (7/14) patients with the non-IGFBP1^hi^WNT3A^lo^ subtype responded, and the difference was statistically significant (*p* = 0.047). We observed long RFS in patients with the IGFBP1^hi^WNT3A^lo^ subtype (median 8.2 vs. 4.9 months, *p* = 0.165) (Figure 5D). Moreover, the results of ROC analysis suggested (Figure 5E) that the IGFBP1^hi^WNT3A^lo^ signature had a better predictive ability than PD-L1 expression (signature AUC = 0.750; PD-L1 AUC = 0.571).

With the aim to evaluate the survival difference between IGFBP1^hi^WNT3A^lo^ subtype patients with or without immunotherapy, we performed a survival analysis in all of the IGFBP1^hi^WNT3A^lo^ patients in the GSE183924 and BJCH cohort, and the results revealed that patients with the IGFBP1^hi^WNT3A^lo^ subtype who received immunotherapy had long RFS (median 25.2 vs. 9.4 months, *p* = 0.026) (Figure 5F).

### 3.7. Characterization of the Immune Cell Infiltration and Immune Features of the IGFBP1^hi^WNT3A^lo^ Subtype

**Figure 6:**
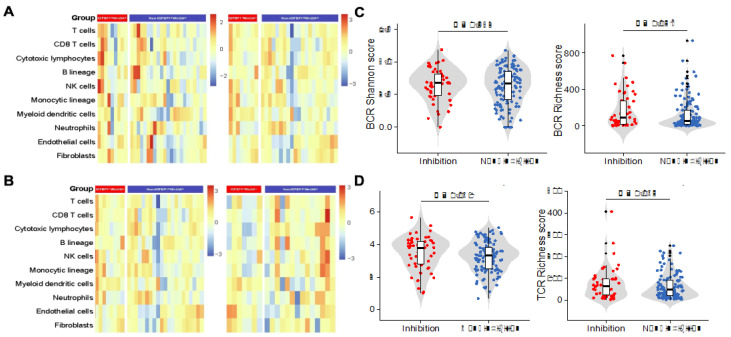
Immune cell infiltration and immunological features. (**A**) Immune cell infiltration in the GSE165252 cohort, shown on the left before immunotherapy and on the right after immunotherapy; (**B**) immune cell infiltration in the GSE165252 cohort and the BJIM cohort, showing the GSE165252 cohort after immunotherapy on the left and the BJIM cohort after immunotherapy on the right; (**C**,**D**) violin plots of BCR/TCR richness and diversity (Shannon score).

We analyzed immune cell infiltration before and after immunotherapy in patients with the IGFBP1^hi^WNT3A^lo^ and non-IGFBP1^hi^WNT3A^lo^ subtypes in the GSE165252 cohort (Figure 6A). Before treatment, there was no significant difference in immune cell infiltration between the two subtypes (*p* > 0.05). After treatment, DC infiltration was significantly increased in the IGFBP1^hi^WNT3A^lo^ subtype (1.80 ± 0.22 vs. 1.50 ± 0.27, *p* = 0.022). Additionally, pre- and post-treatment compassion in the same subtype revealed that increased infiltration of DCs after immunotherapy in the IGFBP1^hi^WNT3A^lo^ subtype (1.65 ± 0.30 vs. 1.80 ± 0.22, *p* = 0.253), though patients with the non-IGFBP1^hi^WNT3A^lo^ subtype showed a significant decrease in DCs infiltration after immunotherapy (1.73 ± 0.34 vs. 1.50 ± 0.27, *p* = 0.043). This trend was validated in the BJIM clinical cohort (Figure 6B), whereby infiltration of DCs was significantly increased in patients with the IGFBP1^hi^WNT3A^lo^ subtype after immunotherapy (1.78 ± 0.24 vs. 1.53 ± 0.30, *p* = 0.047).

Overall, differences in BCR/TCR between the two subtypes may explain the above findings (Figure 6C,D). Compared to the non-IGFBP1^hi^WNT3A^lo^ subtype, the IGFBP1^hi^WNT3A^lo^ subtype had higher BCR richness (189.0 ± 191.3 vs. 130.3 ± 168.4, *p* = 0.038) and TCR richness (81.9 ± 72.3 vs. 63.7 ± 55.4, *p* = 0.063). Despite no significant difference in BCR and TCR diversity between the two subtypes, the same elevated trend was observed in the IGFBP1^hi^WNT3A^lo^ subtype. Significantly downregulated expression levels of genes involved in immune co-suppression were observed in the IGFBP1^hi^WNT3A^lo^ subtype (*p* < 0.05), which included PD-L1/PD-L2 and TGF-β1 expression (Figure S1A–C). In addition, expression levels of genes involved in the antigen presentation process were significantly higher (*p* = 0.020) (Figure S1D), consistent with previous findings (Figure 6). Overall, the immune profile of the IGFBP1^hi^WNT3A^lo^ subtype is consistent with the clinical course during immunotherapy.

## 4. Discussion

Immunotherapy is now the first-line treatment for advanced esophageal cancer. However, there is heterogeneity in patient response to immunotherapy, and most patients do not benefit from it [9]. The biomarkers currently approved by the FDA for clinical use in esophageal cancer are PD-L1 expression (CPS) [4,32] and TMB [33,34]. Unfortunately, the performance of these biomarkers has been inconsistent. In this study, using public databases and our clinical cohorts, we found the genes IGFBP1 and WNT3A from WNT signaling, which are closely associated with immunotherapy for esophageal cancer. Further analysis revealed that the IGFBP1^hi^WNT3A^lo^ signature is associated with the good outcome of immunotherapy in both American and Chinese populations with esophageal cancer, and has better predictive efficacy than PD-L1 expression. In addition, we found that immunotherapy could improve the prognosis of patients with the IGFBP1^hi^WNT3A^lo^ subtype of esophageal cancer. Therefore, we suggest that the IGFBP1^hi^WNT3A^lo^ signature may be used as a biomarker for immunotherapy in patients with esophageal cancer.

IGFBP1 was the first protein in the IGFBP family to be identified and purified, and it regulates the IGF signaling by binding to IGF [35]. Studies have identified IGF-I and IGF-I R as potential biomarkers of prognosis for immunotherapy [36]. As a potential immunotherapeutic target, Wu et al. [37] found that IGF-I R inhibitor combined with oxaliplatin and PD-1 inhibitor significantly inhibited “cold tumor” growth while increasing CTL infiltration and decreasing Treg infiltration within a tumor. Studies investigating the mechanism by which short-term starvation enhances the efficacy of immunotherapy have shown that short-term starvation increases the antitumor response of CD8^+^ T cells by suppressing IGF-I expression, and further analyses have found better immunotherapy efficacy in patients with a lower level of IGF-I and IGF-I R in blood [38].

WNT3A is an important member of the WNT ligand family and activates canonical WNT signaling. Canonical WNT signaling is confirmed to be involved in tumor immune escape in melanoma [15], colorectal cancer [39] and triple-negative breast cancer [40]. Experiments in glioblastoma [41] have shown that blocking canonical WNT signaling enhances CD4^+^/CD8^+^ immune cell infiltration in the TME, dramatically improving sensitivity to PD-1 therapy in gliomas while igniting ‘cold tumors’, suggesting that WNT3A may be a risk factor for immunotherapy. These findings are consistent with our results that IGFBP1^hi^WNT3A^lo^ esophageal cancer patients are more likely to benefit from immunotherapy.

However, we found that esophageal cancer patients with IGFBP1^hi^WNT3A^lo^ do not benefit from conventional standard treatment. In further analysis, we guessed the main reason was that the IGFBP1^hi^WNT3A^lo^ esophageal cancer patients had significantly elevated ITH. ITH is one of the key causes of tumor treatment failure and patient death [42], and studies have confirmed that elevated ITH is associated with poorer prognosis in a variety of solid tumors [43,44]. Another possible explanation was that the stemness features are significantly higher in the IGFBP1^hi^WNT3A^lo^ subtype. The persistence of cancer stem cells (CSCs) confers tumor progression, recurrence, and drug resistance [45]. Early studies proved that high stemness features had shorter OS than patients with low stemness features [46]. These findings may explain the poor prognosis of patients with the IGFBP1^hi^WNT3A^lo^ subtype.

Surprisingly, immunotherapy could improve the survival of IGFBP1^hi^WNT3A^lo^ esophageal cancer patients, and we also found that IGFBP1^hi^WNT3A^lo^ esophageal cancer patients appeared to exhibit immune activation. First, GO enrichment analysis revealed a significant upregulation of immune-related pathways in the IGFBP1^hi^WNT3A^lo^ subtype. This is consistent with the finding of Alzaid et al. [47] that IGFBP1 and IGFBP6 increased proinflammatory cytokine expression when trout were infected with bacteria. Activation of canonical WNT signaling by WNT3A, on the other hand, induces immune escape by significantly upregulating PD-L1 expression [48], while activation of canonical WNT signaling prevents patients with high PD-L1 expression from benefiting from immunotherapy, which may be associated with reduced CD8^+^ T-cell infiltration [49]. In this study, we found that PD-L1/PD-L2 expression was significantly reduced in patients with the IGFBP1^hi^WNT3A^lo^ subtype. In addition, we also found that antigen presentation was significantly enhanced in patients with the IGFBP1^hi^WNT3A^lo^ subtype, as evidenced by a significant increase in the richness and diversity of either BCR or TCR.

Interestingly, the completely opposite change in DCs infiltration before and after immunotherapy between the two subtypes suggests that the IGFBP1^hi^WNT3A^lo^ signature is a biomarker independent of immune cell infiltration and can predict immunotherapy efficacy before treatment. DCs are currently recognized as the most powerful antigen-presenting cells and play a key role in activating antitumor CD8^+^ T-cell immunity [50,51]. Available evidence suggests that the presence of various cytokines in the TME that inhibit the activation of DCs leads to abnormal function of DCs that fail to initiate effective antitumor immunity and even promote tumor progression [52,53]. Studies on fish infections have shown that IGFBP1 and IGFBP6 function similarly and can promote inflammation [47]. In another study, hyperthermia upregulated the expression of IGFBP6 in DCs, which increased the chemotaxis of monocytes and T lymphocytes [54]. These studies indicated that IGFBP1 may regulate the DCs in the TME of ESCA. Meanwhile, canonical WNT signaling regulates DCs’ function at multiple levels. Firstly, canonical WNT and Notch signaling synergistically promote DCs differentiation in humans and mice [55]. Secondly, the decreased secretion of the immune cell chemokine CCL4 in genetically engineered mouse models with conditionally expressing β-catenin resulted in inadequate recruitment of DCs and promoted tumor growth [16]. Finally, the coreceptors LRP5 and LRP6 are key receptors for the activation of canonical WNT signaling, and specific deletion of LRP5 and LRP6 in DCs is associated with delayed tumor progression and enhanced host antitumor immunity [56]. In the present study, the IGFBP1^hi^WNT3A^lo^ subtype appeared to promote immunotherapeutic efficacy in esophageal cancer patients by enhancing the function of DCs. Thus, we assumed that WNT inhibitor may help reverse immunotherapy resistance. DKN-01 is a humanized mAb to anti-DKK1, which has an active role in maintaining an immunosuppressive TME. A phase Ib study of the DKN-01 in combination with pembrolizumab in advanced esophagogastric cancers demonstrated that the combination is well tolerated; signs of clinical activity were also observed, antitumor activity was enriched in anti-PD-(L)1 patients with GEJ/GC whose tumors expressed high DKK1 [57]. We look forward to more clinical trial in the future.

However, our study has some limitations. Firstly, it was a single-center, small sample retrospective study. Secondly, the follow-up of the patients who underwent immunotherapy is short, which could affect the results of the survival analysis. Thirdly, the data from the public database combined adenocarcinoma and squamous cell carcinoma. We need further study to evaluate if our results suit the different histologic esophageal cancer.

## 5. Conclusions

In summary, based on WNT signaling, this study successfully constructed the IGFBP1^hi^WNT3A^lo^ signature and validated its potential as an immunotherapeutic biomarker for esophageal cancer. The signature not only correlated with the prognosis of esophageal cancer patients but also predicted the efficacy of immunotherapy. Analysis of the immune mechanisms showed that enhanced infiltration of DCs allowed esophageal cancer patients to benefit from immunotherapy. Our work improves the understanding of the role of WNT signaling in esophageal cancer immunotherapy response and prognosis, supporting the precise use of an immune agent for esophageal cancer.

## Figures and Tables

**Table 1:** Comparison of clinical characteristics in the BJIM cohort.

Characteristics	Overall, *n* = 21	IGFBP1^hi^WNT3A^lo^, *n* = 7 (%)	Non-IGFBP1^hi^WNT3A^lo^, *n* = 14 (%)	*p* Value
Sex				1.000
Male	19	6 (85.7%)	13 (92.9%)	
Female	2	1 (14.3%)	1 (7.1%)	
Age, years				
<60	12	3 (42.9%)	9 (64.3%)	0.397
≥60	9	4 (57.1%)	5 (35.7%)	
Smoking				1.000
No	5	2 (28.6%)	3 (21.4%)	
Yes	16	5 (71.4%)	11 (78.6%)	
Drinking				0.354
No	6	3 (42.9%)	3 (21.4%)	
Yes	15	4 (57.1%)	11 (78.6%)	
Family history				0.533
No	19	7 (100.0%)	12 (85.7%)	
Yes	2	0 (0.0%)	2 (14.3%)	
Tumor length, cm				0.656
≤3	8	2 (28.6%)	6 (42.9%)	
>3	13	5 (71.4%)	8 (57.1%)	
Differentiation				0.762
Low	8	2 (28.6%)	6 (42.9%)	
Middle	12	5 (71.4%)	7 (50.0%)	
High	1	0 (0.0%)	1 (7.1%)	
AJCC stage				1.000
I+II	1	0 (0.0%)	1 (7.1%)	
III+IV	20	7 (100.0%)	13 (92.9%)	

## Data Availability

Data are available upon reasonable request. Data are available in a public, open access repository.

## Supplementary Materials

The following supporting information can be downloaded at: https://www.mdpi.com/article/10.3390/biology11111575/s1, Figure S1: Immunoregulatory gene expression profile. (A and B) Box plots demonstrating the expression of the immune co-suppressor genes PD-L1/PD-L2; (C) Box plot demonstrating TGF-β1 expression associated with “immune-desert tumor” formation; (D) Box plot demonstrating the expression of the antigen-presenting gene HLA-A; Table S1: Upregulated genes in the Cluster 3 subtype; Table S2: Downregulated genes in the Cluster 3 subtype; Table S3: Correlation analysis of significantly upregulated as well as downregulated genes; Table S4: Comparison of clinical characteristics in the BJCH cohort; Table S5: Multifactorial Cox regression analysis in the BJCH cohort.
