# Supplementary material for: IGFBP1hiWNT3Alo Subtype in Esophageal Cancer Predicts Response and Prolonged Survival with PD-(L)1 Inhibitor
Source: Biology (Basel). 2022 Oct 27;11(11):1575. doi: 10.3390/biology11111575 (PMC9687176; doi:10.3390/biology11111575)
Supplement: Supplementary file 1 [file biology-11-01575-s001.zip › Figure S1. Immunoregulatory gene expression profile.pdf]

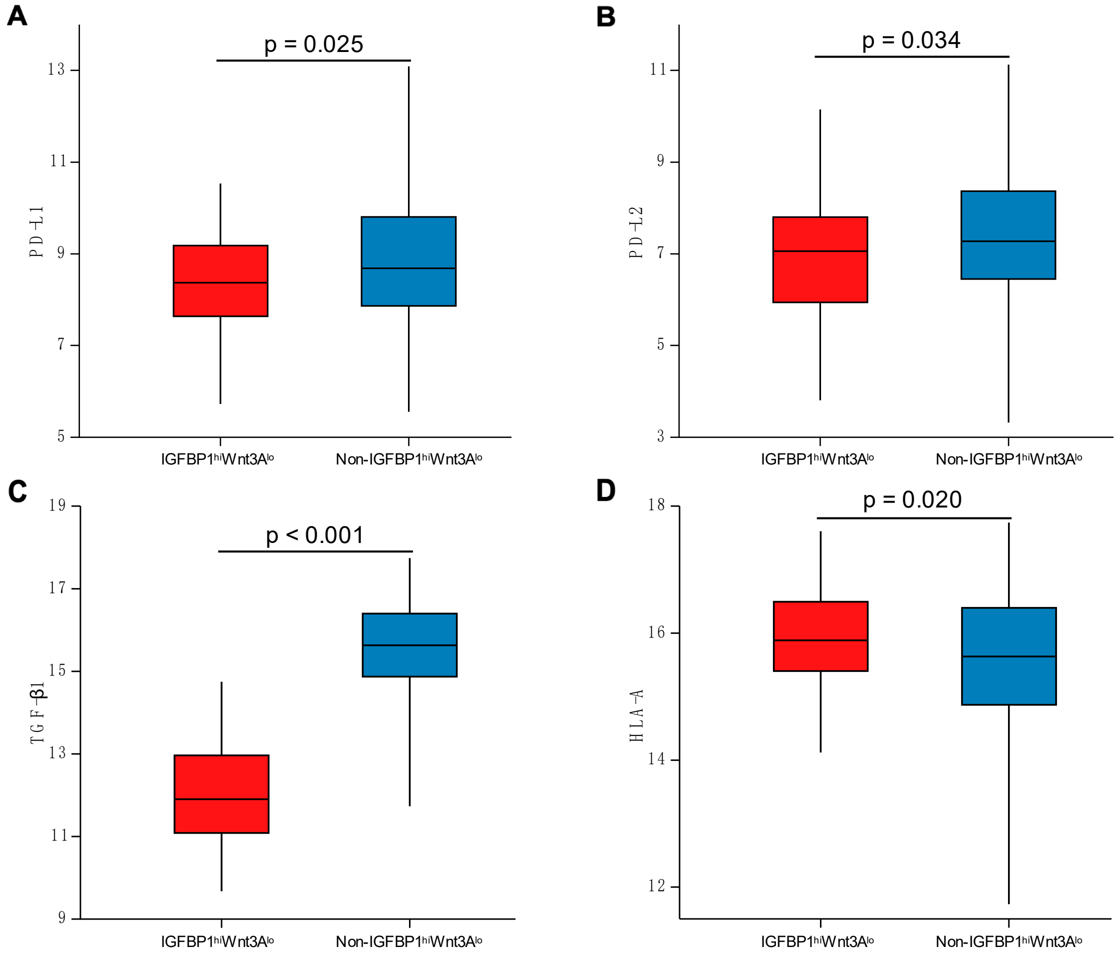

Figure S1. Immunoregulatory gene expression profile. (**A**,**B**) Box plots demonstrating the expression of the immune co-suppressor genes PD-L1/PD-L2. (**C**) Box plot demonstrating TGF-β1 expression associated with "immune-desert tumour" formation. (**D**) Box plot demonstrating the expression of the antigen-presenting gene HLA-A.
