# Supplementary material for: IGFBP1hiWNT3Alo Subtype in Esophageal Cancer Predicts Response and Prolonged Survival with PD-(L)1 Inhibitor
Source: Biology (Basel). 2022 Oct 27;11(11):1575. doi: 10.3390/biology11111575 (PMC9687176; doi:10.3390/biology11111575)
Supplement: Supplementary file 1 [file biology-11-01575-s001.zip › Table S1. Upregulated genes in the Cluster 3 subtype.pdf]

biology

MDPI

Table S1. Upregulated genes in the Cluster 3 subtype.

Gene Symbol	log2(FC)	*P*-value
IGFBP1	4.55	<0.001
AXIN2	1.37	<0.001
PRKCA	0.88	<0.001
ROR1	0.69	0.003
PRKCD	0.56	<0.001
ROCK2	0.51	<0.001
DKK1	0.5	0.114
LRP5	0.49	0.001
LRP6	0.44	<0.001
TLE1	0.43	<0.001
PTPRO	0.41	0.065
DDIT3	0.39	0.002
GNAQ	0.35	<0.001
APOE	0.33	0.181
CTNNB1	0.31	0.001
EDA	0.29	0.182
XIAP	0.29	<0.001
RHOA	0.24	<0.001
MAPK8	0.23	0.011
PPM1B	0.22	<0.001
WNT11	0.2	0.52
ROCK1	0.19	0.032
ATP6AP2	0.18	0.028
C20orf111	0.13	0.129
WNT1	0.12	0.68
UBE2B	0.01	0.921
