# Supplementary material for: IGFBP1hiWNT3Alo Subtype in Esophageal Cancer Predicts Response and Prolonged Survival with PD-(L)1 Inhibitor
Source: Biology (Basel). 2022 Oct 27;11(11):1575. doi: 10.3390/biology11111575 (PMC9687176; doi:10.3390/biology11111575)
Supplement: Supplementary file 1 [file biology-11-01575-s001.zip › Table S2. Downregulated genes in the Cluster 3 subtype.pdf]

biology

MDPI

Table S2. Downregulated genes in the Cluster 3 subtype.

**Gene Symbol**	**log2(FC)**	***P*-value**
WNT3A	-5.6	<0.001
WNT7A	-3.22	<0.001
CAMK2A	-2	<0.001
FAM123A	-1.44	0.03
WNT5A	-1.22	<0.001
SOSTDC1	-1.11	0.002
EGFR	-1.1	<0.001
ROR2	-0.87	<0.001
DAAM1	-0.73	<0.001
APC2	-0.59	<0.001
NFATC1	-0.58	<0.001
CSNK1A1	-0.53	<0.001
JUN	-0.4	0.004
AMFR	-0.4	<0.001
EXT1	-0.4	<0.001
CSNK1G2	-0.32	<0.001
FGFR2	-0.28	0.144
VANGL1	-0.28	0.006
FAM53B	-0.27	0.002
YAP1	-0.22	0.012
LZTS2	-0.21	0.025
DAB2IP	-0.19	0.069
APC	-0.1	0.228
AXIN1	-0.07	0.427
BCL9L	-0.07	0.418
RAC1	-0.03	0.711
CCDC88C	-0.03	0.777
TMEM64	-0.02	0.874
CDC42	-0.01	0.867
