# Supplementary material for: IGFBP1hiWNT3Alo Subtype in Esophageal Cancer Predicts Response and Prolonged Survival with PD-(L)1 Inhibitor
Source: Biology (Basel). 2022 Oct 27;11(11):1575. doi: 10.3390/biology11111575 (PMC9687176; doi:10.3390/biology11111575)
Supplement: Supplementary file 1 [file biology-11-01575-s001.zip › Table S3. Correlation analysis of significantly upregulated as well as downregulated genes.pdf]

biology

MDPI

Table S3. Correlation analysis of significantly upregulated as well as downregulated genes

Gene Set	R (Pearson)	*P*-value
IGFBP1-WNT3A	-0.308	<0.001
IGFBP1-WNT5A	-0.028	0.713
IGFBP1-WNT7A	-0.267	<0.001
IGFBP1-CAMK2A	-0.136	0.0074
AXIN2-WNT3A	-0.311	<0.001
AXIN2-WNT5A	-0.162	0.033
AXIN2-WNT7A	-0.381	<0.001
AXIN2-CAMK2A	-0.064	0.401
PRKCA-WNT3A	-0.548	<0.001
PRKCA-WNT5A	-0.179	0.019
PRKCA-WNT7A	-0.382	<0.001
PRKCA-CAMK2A	-0.033	0.666
PRKCD-WNT3A	-0.369	<0.001
PRKCD-WNT5A	-0.008	0.914
PRKCD-WNT7A	-0.235	0.002
PRKCD-CAMK2A	-0.168	0.027
ROR1-WNT3A	-0.375	<0.001
ROR1-WNT5A	-0.267	<0.001
ROR1-WNT7A	-0.326	<0.001
ROR1-CAMK2A	-0.088	0.251
ROCK2-WNT3A	-0.299	<0.001
ROCK2-WNT5A	-0.019	0.799
ROCK2-WNT7A	-0.265	<0.001
ROCK2-CAMK2A	0.123	0.107
