# Supplementary material for: IGFBP1hiWNT3Alo Subtype in Esophageal Cancer Predicts Response and Prolonged Survival with PD-(L)1 Inhibitor
Source: Biology (Basel). 2022 Oct 27;11(11):1575. doi: 10.3390/biology11111575 (PMC9687176; doi:10.3390/biology11111575)
Supplement: Supplementary file 1 [file biology-11-01575-s001.zip › Table S4. Comparison of clinical characteristics in the BJCH cohort.pdf]

Table S4. Comparison of clinical characteristics in the BJCH cohort.

**Characteristics**	**Overall, n = 95**	**IGFBP1^{hi}WNT3A^{lo}, n = 31(%)**	**Non-IGFBP1^{hi}WNT3A^{lo}, n = 64(%)**	***P*-value**
Sex				0.168
Male	78	28(90.3%)	50(78.1%)	
Female	17	3(9.7%)	14(21.9%)	
Age, years				0.262
＜60	37	15(48.4%)	22(34.4%)	
≥60	58	16(51.6%)	42(65.6%)	
Smoking				0.816
No	31	11(35.5%)	20(31.3%)	
Yes	64	20(64.5%)	44(68.8%)	
Drinking				0.648
No	33	12(38.7%)	21(32.8%)	
Yes	62	19(61.3%)	43(67.2%)	
Family history				0.010*
No	66	27(87.1%)	39(60.9%)	
Yes	29	4(12.9%)	25(39.1%)	
Tumor length, cm				0.268
≤3	55	15(48.4%)	40(62.5%)	
＞3	40	16(51.6%)	24(37.5%)	
Histological type				0.267
Squamous cell carcinoma	87	30(96.8%)	57(89.1%)	
Adenocarcinoma	8	1(3.2%)	7(10.9%)	
Differentiation				0.429
Low	30	10(32.3%)	20(31.3%)	
Middle	55	16(51.6%)	39(60.9%)	
High	10	5(16.1%)	5(7.8%)	
AJCC stage				0.330
I+II	25	6(19.4%)	19(29.7%)	
III+IV	70	25(80.6%)	45(70.3%)	
Treatment				0.804
Non-neoadjuvant	25	9(29.0%)	16(25.0%)	
Neoadjuvant	70	22(71.0%)	48(75.0%)	
