# Supplementary material for: IGFBP1hiWNT3Alo Subtype in Esophageal Cancer Predicts Response and Prolonged Survival with PD-(L)1 Inhibitor
Source: Biology (Basel). 2022 Oct 27;11(11):1575. doi: 10.3390/biology11111575 (PMC9687176; doi:10.3390/biology11111575)
Supplement: Supplementary file 1 [file biology-11-01575-s001.zip › Table S5. Multifactorial Cox regression analysis in the BJCH cohort.pdf]

biology

Table S5. Multifactorial Cox regression analysis in the BJCH cohort.

Characteristics	HR	95%CI		*P*-value
		low	high	
Age	0.681	0.336	1.378	0.285
TNM	0.021	0.180	0.042	0.021*
IGFBP1^{hi}WNT3A^{lo}	2.061	1.012	4.200	0.046*
